# O-GlcNAc transferase couples nutrient availability to synaptic plasticity in paraventricular neurons to regulate satiety

**DOI:** 10.1016/j.jbc.2025.111124

**Published:** 2025-12-30

**Authors:** Mario Pérez-del-Pozo, Manish Bhattacharjee, Anushree Tripathi, Thyra Boafo, Sabrina Galizia, Paolo Medini, Michael Druzin, Olof Lagerlöf

**Affiliations:** 1 Department of Medical Translational Biology, Umeå University, Umeå, Sweden; 2 Department of Clinical Sciences, Umeå University, Umeå, Sweden; 3 Wallenberg Centre for Molecular Medicine, Umeå University, Umeå, Sweden

**Keywords:** O-GlcNAc transferase, satiation, synaptic plasticity, paraventricular nucleus, glucose sensing, feeding behavior, neuronal excitability

## Abstract

Satiation is essential for energy homeostasis and is dysregulated in metabolic disorders like obesity and eating disorders such as anorexia nervosa. While satiation engages a large neural network across brain regions, how the communication within this network depends on metabolic fluctuations is unclear. This study shows that nutrient access can affect neuron-to-neuron communication in this network by regulating excitatory synaptic plasticity through O-GlcNAc transferase (OGT) in αCaMKII satiation neurons in the paraventricular nucleus (PVN). Using cell-specific knockout mice and electrophysiological recordings, we demonstrate that OGT deletion in PVN^αCaMKII^ neurons increases input resistance and neuronal excitability while preserving basic membrane electrical properties. Strikingly, feeding triggered a robust 3.8-fold increase in excitatory synaptic input in wild-type neurons, whereas OGT-knockout neurons failed to exhibit this feeding-induced synaptic activation and instead displayed a paradoxical trend towards decreased synaptic activity upon food intake. Furthermore, OGT deletion destabilized glucose-dependent synaptic responses, with knockout neurons displaying maladaptive depression of excitatory transmission in conditions where stability is normally preserved. These findings establish OGT as a nutrient-sensitive modulator of synaptic plasticity that ensures appropriate satiation signaling by coupling metabolic state to synaptic plasticity.

Satiation is the process that occurs during eating and leads to meal termination, providing the physiological signal to stop consuming the current meal. It is a fundamental and complex physiological process critical for energy homeostasis. It is regulated by a network of neural and hormonal signals (1, 2). Many of these signals affect food intake through the hypothalamus in the brain. The paraventricular nucleus (PVN) of the hypothalamus plays a fundamental role in feeding behavior by integrating peripheral signals of energy status with emotional and cognitive aspects of feeding behavior (3, 4, 5). Some of its neurons communicate with other parts of the brain through direct neuron-to-neuron contacts, or synapses, while other neurons are part of the neuroendocrine system and affect body metabolism by sending processes to the pituitary and median eminence. Disruption of these mechanisms can lead to dysregulated eating patterns and contribute to metabolic disorders such as obesity, as well as eating disorders, including anorexia nervosa (6), highlighting the importance of understanding how their function is coupled to the metabolic state of the body.

Central to this metabolic coupling is the brain's ability to monitor glucose availability through specialized glucose-sensing mechanisms within hypothalamic circuits (7, 8). The most well-characterized system is the classical GLUT2/glucokinase/K_ATP_ channel pathway that creates glucose-excited and glucose-inhibited neurons analogous to pancreatic β-cells (9, 10, 11). These threshold-based sensors respond to glucose concentrations within their physiological range and have proven essential for basic glucose homeostasis, particularly in counterregulatory responses to hypoglycemia. They are either turned on or turned off, respectively, by glucose and in response regulate intrinsic excitability of the neuron.

In addition to these established pathways, a significant fraction of nutrients taken up by cells is metabolized through the hexosamine biosynthesis pathway (HBP) to uridine-diphosphate N-acetylglucosamine (UDP-GlcNAc). UDP-GlcNAc is used as a donor substrate by the enzyme O-GlcNAc transferase (OGT). OGT catalyzes the transfer of β-N-acetylglucosamine (GlcNAc) to serine and threonine residues of intracellular proteins (O-GlcNAc) (12). O-GlcNAc can then be removed by O-GlcNAcase (OGA). Its dependence on flux through the HBP and regulation by other metabolic pathways, such as insulin, suggest that OGT regulates cellular function depending on the body’s metabolic status (13). Indeed, the HBP and O-GlcNAc cycling are linked genetically to body weight and metabolic disorders in humans (13, 14, 15, 16). We have previously shown that OGT can regulate food intake. In the PVN, O-GlcNAc levels are sensitive to metabolic fluctuations, particularly in αCaMKII-expressing cells (PVN^αCaMKII^) (17). While these neurons have not been fully characterized, our previous observations show that these neurons become activated upon food intake. Once activated, as revealed by optogenetics, they turn off further food intake (5). This feeding-induced activation is completely dependent on OGT. Deleting OGT in these neurons during a time window where there is no apparent effect on cell health increases food intake and body weight rapidly due to a larger meal size. It may be the case that this effect on energy homeostasis is compensated for by other neurons after several months (17, 18, 19, 20). Furthermore, αCaMKII-expressing neurons in certain brain regions have been shown to suppress further food intake upon activation, as demonstrated in the anterior insular cortex, pointing to a broader role for these neurons in feeding regulation (21). These and other data indicate that the PVN^αCaMKII^ neurons mediate satiation, at least in part through OGT. We and others have reported that OGT can regulate neuron function by affecting excitatory neurotransmission: OGT regulates both excitatory synapse number and synapse strength in part by controlling the synaptic abundance of AMPA receptors (17, 22). It has also been shown also that glutamatergic neurotransmission through the AMPA receptor in the PVN regulates food intake (22, 23). However, unlike classical glucose sensors that exhibit binary responses to acute nutrient changes, it is unclear whether and how OGT may affect excitatory neurotransmission in PVN^αCaMKII^ neurons in a nutrient-dependent manner to regulate satiation.

The current study investigates the electrophysiological mechanisms through which OGT regulates PVN^αCaMKII^ neuronal function under varying metabolic conditions. We demonstrate that selective deletion of OGT from PVN^αCaMKII^ increases neuronal input resistance and increases excitability while preserving basic membrane electrical properties. Most strikingly, we identify a critical role for OGT in coupling nutritional status to synaptic transmission, as OGT-knockout (OGT-KO) neurons exhibit a paradoxical decline in excitatory synaptic input during feeding—the opposite pattern observed in wild-type (WT) neurons, where feeding strongly enhances excitatory synaptic input. Furthermore, we demonstrate that OGT maintains robust synaptic responses during acute glucose fluctuations, with OGT-KO neurons displaying marked synaptic depression when exposed to elevated glucose. Taken together, these findings indicate that OGT couples nutrient availability to satiation by regulating excitatory synaptic plasticity in PVN^αCaMKII^.

## Results

### PVN^αCaMKII^ neurons are parvocellular

**Figure 1:**
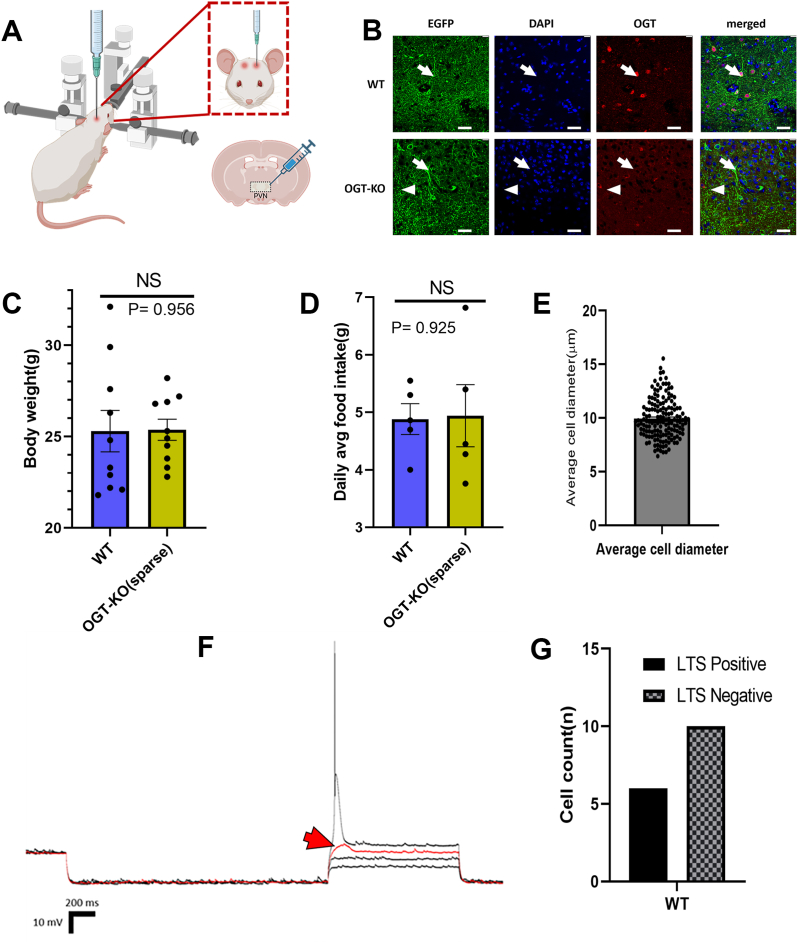
**OGT-KO in the PVN^αCaMKII^ predominantly targets parvocellular neurons.***A*, schematic of stereotactic injection procedure in PVN of OGT-floxed and WT C57BL/6 mice. *B*, Representative confocal micrographs showing EGFP expression (*green*) and OGT immunolabeling (*red*) in WT and OGT-KO brain slices. DAPI counterstains nuclei (*blue*). *White arrows* indicate EGFP-positive neurons in the PVN. *White arrowheads* point to OGT-positive cells that do not express EGFP. In WT sections, *arrows* show neurons expressing both EGFP and OGT (visible in merged image as co-localization). In OGT-KO sections, *arrows* indicate EGFP-positive neurons with reduced or absent OGT immunoreactivity confirming successful knockout, while *arrowheads* show that OGT expression remains in non-targeted cells. Scale bar: 5 μm (applies to all images). *C*, average body weight over 10-day post-surgical period comparing WT vs OGT-KO mice (NS, *p* = 0.956). *D*, daily average food intake over same period comparing genotypes (NS, *p* = 0.925). *E*, cell diameter analysis of EGFP-expressing neurons showing mean diameter of 10.79 μm ± 0.206 μm (SEM); error bars overlap with individual datapoints. *F*, representative current-clamp trace showing Low-threshold spike (LTS) detection protocol in WT neurons. The red sample trace is representative of the LTS; the *red arrowhead* points at the tiny spike observed characteristic feature of LTS. *G*, LTS classification showing 6/16 (37.5%) LTS-positive and 10/16 (62.5%) LTS-negative WT neurons. Data represent recordings from individual neurons across 10 mice: 6 LTS-positive and 10 LTS-negative cells. Data from OGT-floxed (n = 10) and WT (n = 10) mice, except (*panel E*) where OGT-floxed (n = 5) and WT (n = 5) mice were used were used. Cell diameter measured from 153 EGFP-expressing cells. Statistical comparisons using Mann-Whitney test. Data presented as mean ± SEM. Blue bars: WT; yellow bars: OGT-KO.

While the PVN^αCaMKII^ neurons are critical for satiation, their basic morphological and electrophysiological properties have not been characterized. To selectively label and delete OGT from PVN^αCaMKII^ neurons, we utilized stereotactic injections of a cocktail of two AAV1 vectors encoding CaMKII-Cre and floxed enhanced green fluorescent protein (EGFP), respectively, into the PVN of both OGT-floxed and wild-type (WT) C57BL/6 mice (Fig. 1*A*). Immunohistochemical analysis *via* confocal microscopy confirmed robust transduction as evidenced by intense EGFP expression localized within the PVN. We used low-titer viral stocks to achieve sparse OGT deletion in a limited population of PVN^αCaMKII^ neurons. This preserves overall network function and ensures that subsequent electrophysiological analyses reflect cell-autonomous effects in the targeted neurons rather than secondary effects from hyperphagia-induced obesity. OGT immunoreactivity was largely absent in EGFP-labeled KO-neurons, yet residual OGT signal remained in other cells in the PVN, reflecting our deliberately sparse knockout strategy designed to isolate cell-autonomous effects without inducing secondary systemic changes (Fig. 1*B*). We confirmed whether sparse OGT deletion influenced systemic parameters: There was no significant difference in average body weight between WT and OGT-KO mice (WT: 25.3 ± 1.11 g; OGT-KO: 25.37 ± 0.54 g; *p* = 0.956) (Fig. 1*C*), nor in daily food intake (WT: 4.88 ± 0.29 g; OGT-KO: 4.94 ± 0.53 g; *p* = 0.925) (Fig. 1*D*). Our previous observations show that PVN^αCaMKII^ overlap with markers for both thyrotropin-releasing hormone (TRH) and oxytocin neurons (17). TRH is specifically produced by parvocellular neurons (22), while oxytocin can be produced by both parvocellular and magnocellular neurons in the PVN (22, 24, 25). However, quantitative morphometric analysis of 153 EGFP-positive neurons revealed a mean cell diameter of 10.79 μm (±0.206 μm SEM), consistent with the expected size distribution of only parvocellular neurons (Fig. 1*E*). Parvocellular neurons that express a low-threshold spike (LTS) typically send axons to other parts of the brain rather than directly regulating peripheral metabolism through the neurosecretory system in the pituitary (26). A substantial fraction of PVN^αCaMKII^ neurons (6/16; 37.5%) exhibit LTS (Fig. 1, *F* and *G*), marking this as the first report of LTS in this genetically defined population in the PVN. LTS-positive neurons were less frequent than LTS-negative neurons, consistent with their known physiological distribution within the PVN. These findings indicate that PVN^αCaMKII^ mediate satiation at least in part through participating in a larger intra-brain neural network.

### OGT-KO in the PVN^αCaMKII^ does not alter basic electrophysiological properties but increases neuronal excitability

**Figure 2:**
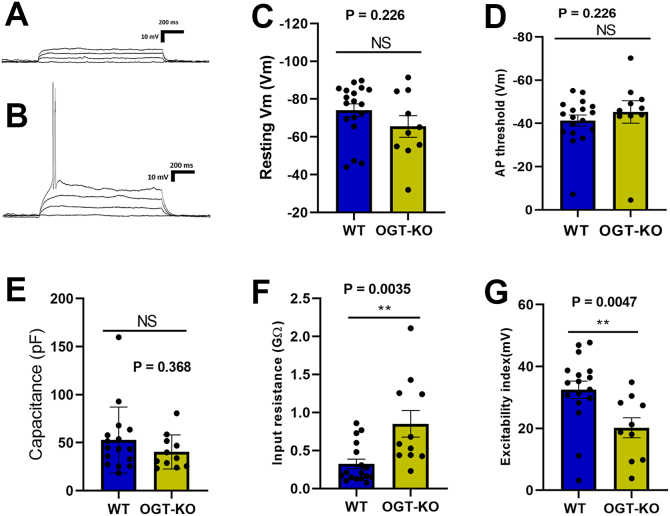
**OGT-KO in the PVN^αCaMKII^ alters input resistance and neuronal excitability without altering basic electrophysiological properties.** Representative current-clamp traces for action potential threshold and baseline membrane potential measurements in (*A*) WT and (*B*) OGT-KO neurons. *C*, resting membrane potential comparing WT vs OGT-KO neurons (NS, *p* = 0.226). *D*, action potential threshold comparing genotypes (NS, *p* = 0.226). *E*, membrane capacitance comparing genotypes (NS, *p* = 0.368). *F*, input resistance comparing WT vs OGT-KO neurons (*p* = 0.0035). *G*, excitability index comparing genotypes (*p* = 0.0047). Data from WT (n = 17–18) and OGT-KO (n = 10–11) PVN parvocellular neurons. Statistical comparisons using Mann–Whitney non-parametric test. Data presented as mean ± SEM. Blue bars: WT neurons; yellow bars: OGT-KO neurons. ∗∗*P* < 0.01, NS, non-significant.

Basic electrophysiological properties were measured to determine whether OGT deletion affects the intrinsic biophysical characteristics of PVN^αCaMKII^ neurons. Representative traces from WT and OGT-KO neurons during AP threshold and baseline membrane potential measurements are shown in Figure 2, *A* and *B*, respectively. Resting membrane potential (WT: −74.02 ± 14.68 mV; OGT-KO: −65.52 ± 18.09 mV; *p* = 0.226), action potential(AP) threshold (WT: −41.32 ± 10.87 mV; OGT-KO: −45.31 ± 16.42 mV; *p* = 0.226), and membrane capacitance (WT: 52.77 ± 34.29 pF; OGT-KO: 40.38 ± 17.77 pF; *p* = 0.368) were not significantly different between WT and OGT-KO neurons (Fig. 2, *C*–*E*). These observations show that deleting OGT does not affect basic neuron properties in PVN^αCaMKII^.

In contrast, properties related to neuronal responsiveness were significantly altered in OGT-KO neurons. Input resistance was markedly increased in OGT-KO neurons (WT: 0.32 ± 0.26 GΩ; OGT-KO: 0.85 ± 0.58 GΩ; *p* = 0.0035) (Fig. 2*F*). The increase in input resistance in OGT-KO neurons implies that even small current inputs will result in larger changes in membrane voltage, effectively amplifying synaptic input. To further characterize the functional consequences of altered input resistance on neuronal excitability, we next examined the threshold for action potential generation by measuring the excitability index. The excitability index is defined as the minimum depolarization required to elicit an AP. Consistent with the increased input resistance, OGT-KO neurons showed a significantly lower excitability index compared to WT neurons (WT: 32.47 ± 11.49 mV; OGT-KO: 20.21 ± 10.23 mV; *p* = 0.0047) (Fig. 2*G*), meaning they required less current injection to fire action potentials and were therefore more excitable (27). These complementary findings demonstrate that OGT deletion enhances the ability of neurons to generate action potentials in response to smaller stimuli, indicating that OGT regulates neuronal excitability in PVN^αCaMKII^ (28).

### OGT regulates feeding-dependent changes in excitatory synaptic input *via* AMPA receptors

**Figure 3:**
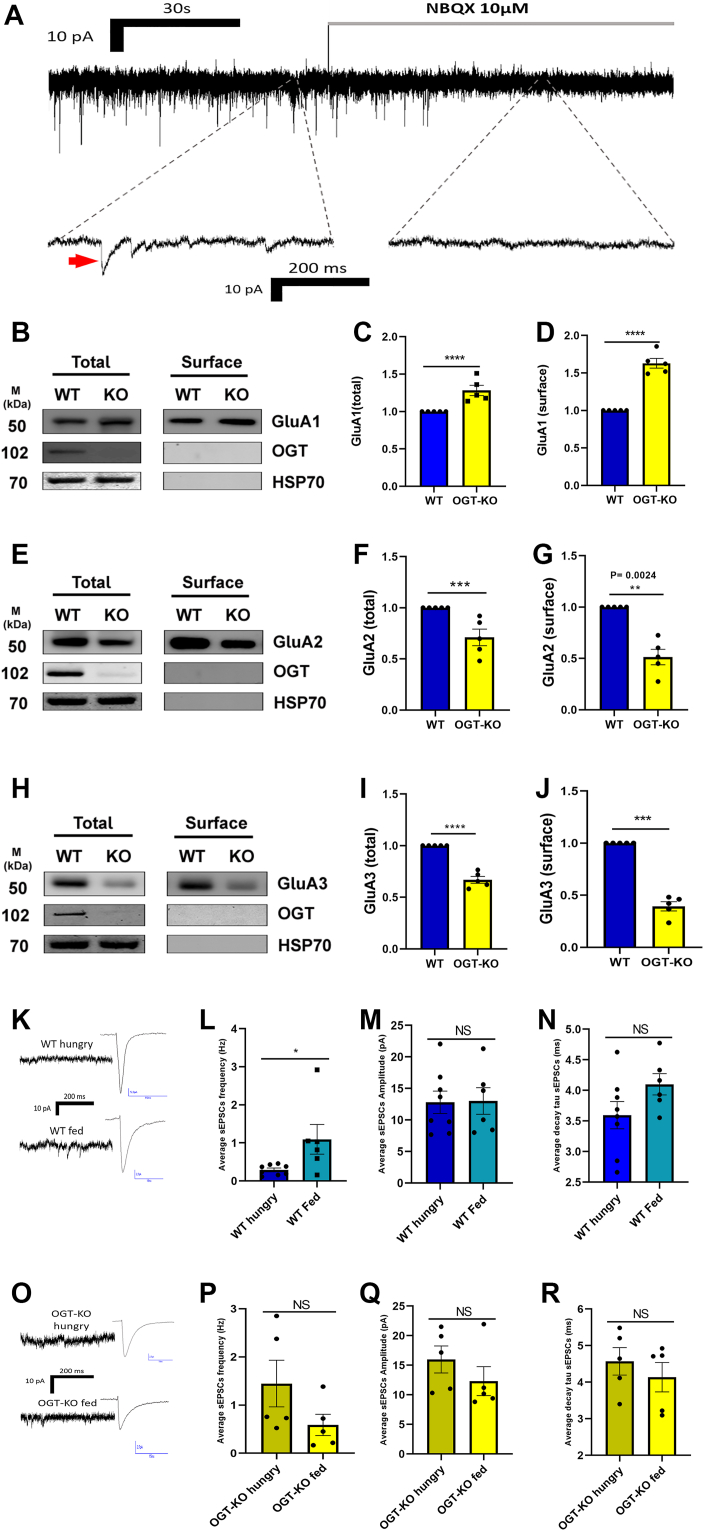
**Nutritional state differentially modulates synaptic transmission in WT and OGT-KO neurons.***A*, representative trace showing suppression of spontaneous excitatory synaptic events following NBQX application in WT PVN^αCaMKII^ neurons. The *red arrow* represents a typical excitatory spike. *B*–*D*, representative Western blots and quantification of total and surface GluA1 expression in primary cortical neurons. WT values were normalized to 1. OGT-KO total GluA1 = 1.282 ± 0.068; surface GluA1 = 1.629 ± 0.064. *E–G*, representative Western blots and quantification of total and surface GluA2 expression. WT values were normalized to 1. OGT-KO total GluA2 = 0.7101 ± 0.081; surface GluA2 = 0.5138 ± 0.075. *H–J*, representative Western blots and quantification of total and surface GluA3 expression. WT values were normalized to 1. OGT-KO total GluA3 = 0.6691 ± 0.032; surface GluA3 = 0.3935 ± 0.044. Western blot quantitative analyses were performed using the Wilcoxon signed-rank test. All Western blot data expressed as mean ± SEM. *K*, representative sEPSC traces in WT neurons under hungry (*top*) and fed (*bottom*) conditions. *L*, sEPSC frequency in WT neurons comparing hungry vs fed states (*p* = 0.021). *M*, sEPSC amplitude in WT neurons comparing hungry vs fed states (NS, *p* = 0.851). *N*, sEPSC decay tau in WT neurons comparing hungry vs fed states (NS, *p* = 0.142). *O*, Representative sEPSC traces in OGT-KO neurons under hungry (*top*) and fed (*bottom*) conditions. *P*, sEPSC frequency in OGT-KO neurons comparing hungry vs fed states (NS, *p* = 0.095). *Q*, sEPSC amplitude in OGT-KO neurons comparing hungry vs fed states (NS, *p* = 0.309). *R*, sEPSC decay tau in OGT-KO neurons comparing hungry vs fed states (NS, *p* = 0.547). All sEPSC data (*panelsK–R*) were obtained from WT (n = 5) and OGT-KO (n = 5) mice under hungry and fed conditions (n = 5 each condition). Statistical comparisons using Mann-Whitney non-parametric test. Data presented as mean ± SEM. *Blue* bars: WT neurons (*dark blue* = hungry, *light blue* = fed); *yellow* bars: OGT-KO neurons (*light yellow* = hungry, *bright yellow* = fed). ∗*P* < 0.05, ∗∗*P* < 0.01, ∗∗∗*P* < 0.001, ∗∗∗∗*P* < 0.0001, NS, non-significant.

Our previous work showed that O-GlcNAc levels in PVN^αCaMKII^ cells decrease during fasting and that OGT regulates excitatory synaptic function (17, 22). To verify that the synaptic events analyzed in this study arise from AMPA receptor-mediated transmission, we first applied the AMPA receptor antagonist NBQX to WT PVN^αCaMKII^ neurons (29). Bath application of NBQX abolished spontaneous synaptic events, confirming that the sEPSCs recorded throughout this study are AMPA receptor dependent (Fig. 3*A*).

We next examined whether OGT deletion alters AMPA receptor trafficking by quantifying total and surface protein levels of GluA1, GluA2 and GluA3 in primary cortical neuron cultures. For all Western blot quantifications, WT values were normalized to 1, and OGT-KO expression levels are reported relative to this baseline. OGT-KO neurons displayed a significant increase in both total GluA1 (OGT-KO: 1.282 ± 0.068; *p* < 0.0001) and surface GluA1 expression (OGT-KO: 1.629 ± 0.064; *p* < 0.0001) compared to WT (Fig. 3, *B*–*D*). In contrast, GluA2 levels were significantly reduced in OGT-KO neurons, with decreased total GluA2 (OGT-KO: 0.7101 ± 0.081; *p* = 0.0009) and reduced surface GluA2 expression (OGT-KO: 0.5138 ± 0.075; *p* = 0.0024) (Fig. 3, *E*–*G*). Similarly, GluA3 abundance was decreased in OGT-KO neurons, with significant reductions in total (OGT-KO: 0.6691 ± 0.032; *p* < 0.0001) and surface fractions (OGT-KO: 0.3935 ± 0.044; *p* = 0.0009) (Fig. 3, *H*–*J*). These results indicate that OGT deletion leads to a compositional change of AMPA receptor subunits, favoring elevated GluA1 and reduced GluA2/3 expression at the neuronal surface.

Given these subunit-specific changes in AMPA receptor composition, we next asked whether OGT deletion alters how PVN^αCaMKII^ neurons function under physiological metabolic conditions. To reveal how OGT influences the response of these neurons to different nutritional states, we recorded spontaneous excitatory postsynaptic currents (sEPSCs) from WT and OGT-KO neurons obtained from mice under hungry and fed conditions (Fig. 3). In WT neurons from fed mice, sEPSC frequency was robustly elevated (1.10 ± 0.95 Hz) compared to neurons from hungry mice (0.29 ± 0.13 Hz; *p* = 0.021) (Fig. 3*L*). This demonstrates that feeding activates excitatory input to PVN^αCaMKII^ neurons, with fed animals showing a nearly 3.8-fold increase in synaptic drive that supports satiation signaling and meal termination.

In contrast, OGT-KO neurons exhibited an opposite trend, with feeding resulting in more than a two-fold lower sEPSC frequency than in the hungry state, although this difference did not reach statistical significance (OGT-KO hungry: 1.45 ± 1.08 Hz; OGT-KO fed: 0.59 ± 0.50 Hz; *p* = 0.095) (Fig. 3*P*).

The amplitude of sEPSCs was not significantly affected by nutritional state in either WT (WT hungry: 12.81 ± 5.00 pA; WT fed: 13.01 ± 5.16 pA; *p* = 0.851) or OGT-KO neurons (OGT-KO hungry: 15.98 ± 5.10 pA; OGT-KO fed: 12.31 ± 5.46 pA; *p* = 0.309) (Fig. 3, *M* and *Q*). Similarly, the decay tau of sEPSCs remained consistent across nutritional states in both genotypes (WT hungry: 3.59 ± 0.63 ms; WT fed: 4.10 ± 0.43 ms; *p* = 0.142; OGT-KO hungry: 4.57 ± 0.84 ms; OGT-KO fed: 4.14 ± 0.90 ms; *p* = 0.547) (Fig. 3, *N* and *R*), indicating that OGT specifically regulates synaptic event frequency rather than strength over periods of longer nutrient fluctuations.

These results suggest that OGT plays a critical role in regulating the frequency of excitatory synaptic input to PVN^αCaMKII^ neurons in response to the feeding state, while not affecting event amplitude or kinetics. In WT neurons, excitatory drive increases upon feeding, consistent with a satiety-promoting mechanism (17). In contrast, OGT-KO neurons have higher excitatory input during fasting and lack feeding-induced enhancement, indicating that OGT is necessary for proper state-dependent modulation of synaptic activity.

### OGT stabilizes synaptic responses to acute glucose fluctuations

Since OGT activity depends on UDP-GlcNAc derived from glucose metabolism and is implicated in nutrient sensing and feeding regulation (30)—we hypothesized that OGT may regulate neuronal responses to acute changes in glucose levels during a time course similar to postprandial changes in glucose concentration.

**Figure 4:**
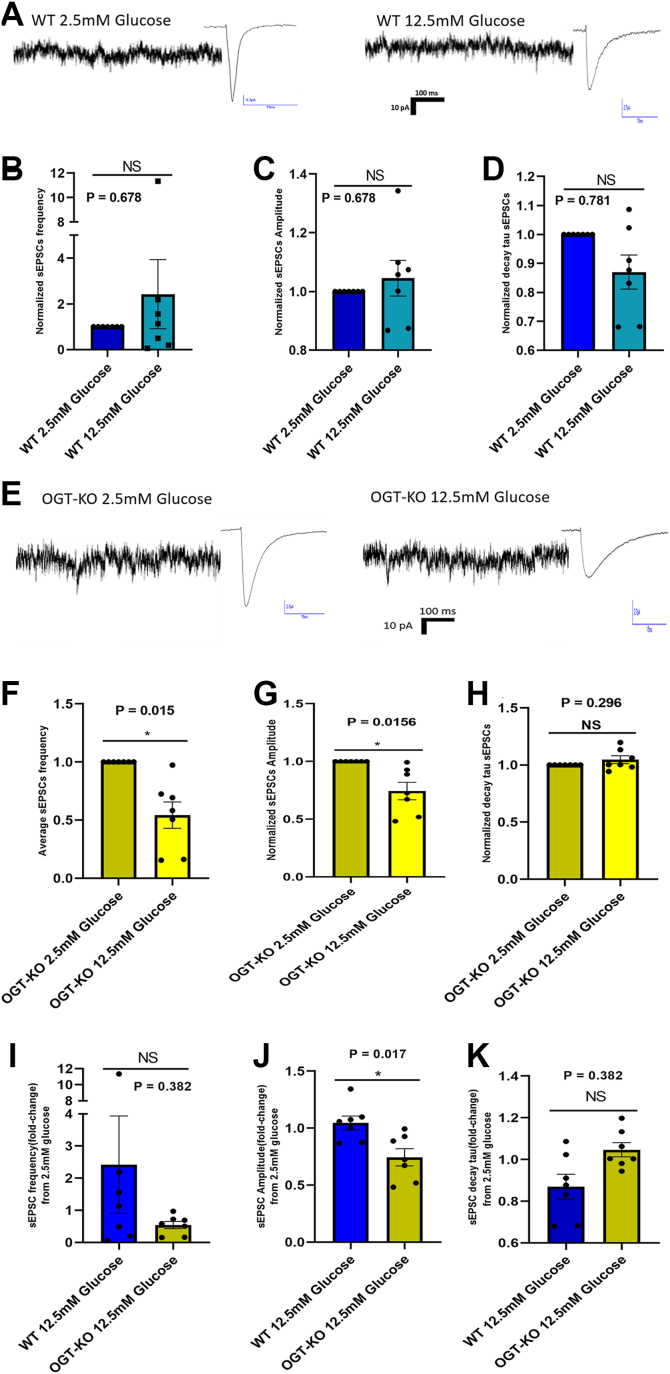
**OGT stabilizes synaptic responses to acute glucose fluctuations.***A*, representative sEPSC traces in WT neurons under 2.5 mM glucose (*left*) and 12.5 mM glucose (*right*). *B*, normalized sEPSC frequency in WT neurons comparing 2.5 mM to 12.5 mM glucose (NS, *p* = 0.687). *C*, normalized sEPSC amplitude in WT neurons comparing 2.5 mM to 12.5 mM glucose (NS, *p* = 0.687). *D*, normalized sEPSC decay tau in WT neurons comparing 2.5 mM to 12.5 mM glucose (NS, *p* = 0.0781). *E*, representative sEPSC traces in OGT-KO neurons under 2.5 mM glucose (*left*) and 12.5 mM glucose (*right*). *F*, normalized sEPSC frequency in OGT-KO neurons comparing 2.5 mM to 12.5 mM glucose (*p* = 0.0156). *G*, normalized sEPSC amplitude in OGT-KO neurons comparing 2.5 mM to 12.5 mM glucose (*p* = 0.0156). *H*, normalized sEPSC decay tau in OGT-KO neurons comparing 2.5 mM to 12.5 mM glucose (NS, *p* = 0.296). Fold-change analyses (12.5 mM relative to 2.5 mM glucose): (*I*) sEPSC frequency fold-change between WT and OGT-KO neurons (NS, *p* = 0.382). *J*, sEPSC amplitude fold-change between WT and OGT-KO neurons (*p* = 0.017). *K*, sEPSC decay tau fold-change between WT and OGT-KO neurons (NS, *p* = 0.382). Data from WT (n = 7) and OGT-KO (n = 7) mice. Normalized values in B-H set 2.5 mM glucose as baseline (=1). Panels B-H analyzed using Wilcoxon signed-rank test; panels I-K analyzed using Mann-Whitney test. Data presented as mean ± SEM. Blue bars: WT neurons; yellow bars: OGT-KO neurons. ∗*P* < 0.05, NS, non-significant.

To directly examine how OGT mediates the neuronal response to glucose availability, we exposed acute brain slices to sequential glucose concentrations of 2.5 mM followed by 12.5 mM (Fig. 4).

In WT neurons, acute elevation of glucose from 2.5 mM to 12.5 mM did not significantly affect normalized sEPSC frequency (12.5 mM Glucose = 2.42 ± 4.0; *p* = 0.687) (Fig. 4*B*), amplitude (12.5 mM Glucose = 1.05 ± 0.16; *p* = 0.687) (Fig. 4*C*), or decay tau (12.5 mM Glucose = 0.87 ± 0.16; *p* = 0.0781) (Fig. 4*D*). This suggests that WT PVN^αCaMKII^ neurons maintain stable synaptic properties during acute glucose fluctuations (18).

In marked contrast, OGT-KO neurons exhibited a significant decrease in synaptic transmission when exposed to elevated glucose. sEPSC frequency markedly decreased in OGT-KO neurons at 12.5 mM glucose (12.5 mM Glucose = 0.54 ± 0.3; *p* = 0.0156) (Fig. 4*F*). Similarly, sEPSC amplitude was significantly reduced in OGT-KO neurons under high glucose conditions (12.5 mM Glucose = 0.74 ± 0.20; *p* = 0.0156) (Fig. 4*G*), while decay tau remained unchanged (12.5 mM Glucose = 1.05 ± 0.09; *p* = 0.296) (Fig. 4*H*). This differential response to acute glucose fluctuations provides evidence that OGT functions to stabilize excitatory synaptic input during metabolic fluctuations, adapting the PVN^αCaMKII^ response to glucose elevations rather than being necessary to detect changes in glucose fluctuations.

When comparing the fold-change responses (12.5 mM Glucose/2.5 mM Glucose) between WT and OGT-KO neurons, we observed that the frequency response did not reach statistical significance (WT: 2.42 ± 4.00; OGT-KO: 0.54 ± 0.30; *p* = 0.382) (Fig. 4*I*), likely due to high variability in WT neurons. However, the amplitude response was significantly different (WT: 1.05 ± 0.16; OGT-KO: 0.74 ± 0.20; *p* = 0.0175) (Fig. 4*J*). The comparison of decay tau fold-changes between the groups showed a trend toward significance (WT: 0.87 ± 0.16; OGT-KO: 1.05 ± 0.09; *p* = 0.053), although it did not reach the conventional threshold for statistical significance (Fig. 4*K*). These results further substantiate that OGT regulates glucose-dependent synaptic plasticity in PVN^αCaMKII^ neurons.

**Figure 5:**
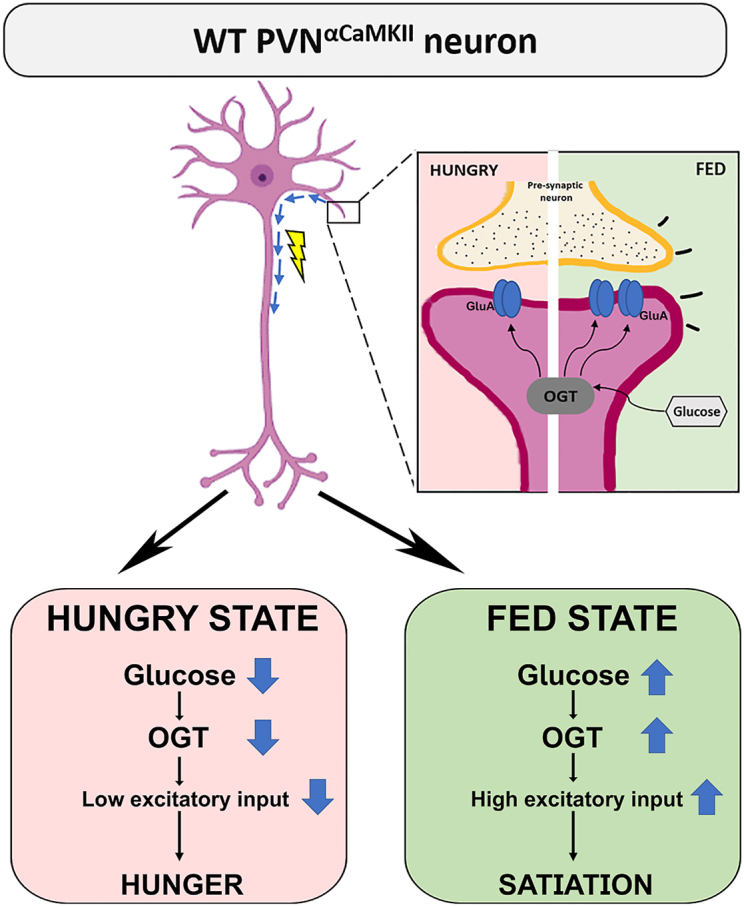
**OGT functions as an adapter of excitatory synaptic plasticity in PVN^αCaMKII^ neurons across nutritional and feeding states.** Schematic summarizing the role of OGT's role as a postsynaptic adapter linking metabolic state to synaptic plasticity in feeding circuits across hungry (*pink* background) and fed (*green* background) states. *Top*: The enlarged inset depicts the synaptic interface where the pre-synaptic terminal (*yellow*) releasing neurotransmitters onto post-synaptic receptors in the PVN^αCaMKII^ neuron (*magenta*). OGT serves as a critical glucose sensor within the post-synaptic compartment, where glucose availability modulates OGT activity and subsequent O-GlcNAcylation of synaptic proteins, thereby controlling synaptic strength and stability. AMPA receptors (GluA) at the postsynaptic site are depicted as the *blue* double oval. *Bottom panels*: In the hungry state (*left*), decreased glucose levels lead to reduced OGT activity, resulting in diminished excitatory synaptic input to promote hunger signaling. Conversely, in the fed state (*right*), elevated glucose enhances OGT function, increasing excitatory synaptic transmission and promoting satiation signaling.

These findings favor a model in which OGT regulates excitatory synaptic input to PVN^αCaMKII^ neurons in response to metabolic fluctuations. In wild-type neurons, excitatory input appropriately increases upon feeding and support satiation, whereas OGT-KO neurons exhibit paradoxical synaptic responses to metabolic fluctuations (31) (Fig. 5). Together, our data show that OGT adapts synaptic plasticity to nutritional status in PVN feeding circuits.

## Discussion

This study demonstrates that OGT in PVN^αCaMKII^ satiation neurons adapts excitatory synaptic transmission to fluctuations in energy substrate availability. Deletion of OGT increases neuronal input resistance and excitability while preserving basic electrophysiological properties (Figs. 1 and 2), and these intrinsic changes are accompanied by a disrupted ability to modulate AMPA receptor-dependent excitatory synaptic input according to feeding status (Fig. 3). Furthermore, OGT is necessary for synaptic responses to acute fluctuations in extracellular glucose, as evidenced by a pronounced synaptic depression in the OGT-KO neurons exposed to high glucose (Fig. 4). These data suggest that OGT mediates satiation by coordinating neuron excitability and excitatory synaptic plasticity in response to nutrient availability. In contrast to the classical GLUT2/glucokinase/K_ATP_ system, our observations favor a model where OGT links satiation not only to intrinsic neuron excitability but makes satiation dependent on neuronal input from other brain regions to the PVN (Fig. 5).

PVN^αCaMKII^ neurons exhibit characteristics consistent with parvocellular network-integrating neurons rather than classical neuroendocrine cells, aligning with their dependence on upstream synaptic input for metabolic regulation (Fig. 1, *F* and *G*) (17). Sparse OGT deletion ensured that the observed electrophysiological effects were cell-autonomous and not secondary to hyperphagia or obesity, allowing direct assessment of how OGT influences neuronal physiology (Figs. 1 and 2). Consistent with findings in AgRP neurons and hippocampus, OGT deletion did not alter basic membrane properties but increased input resistance and firing responsiveness (Fig. 2, *A*–*E*) (32, 33), indicating that OGT modulates how PVN^αCaMKII^ neurons respond to synaptic excitation (34).

OGT was also required for appropriate modulation of excitatory input across nutritional states. In wild-type neurons, feeding enhanced synaptic drive (Fig. 3*L*), whereas this adjustment was absent in OGT-knockout neurons, which instead displayed lower excitatory input in the fed state (Fig. 3*P*). Since excitability was elevated in OGT-KO neurons (Fig. 2, *B*–*E*), this disruption reflects altered synaptic regulation rather than impaired spike generation, consistent with reports that PVNαCaMKII neurons normally receive enhanced excitatory drive during feeding (17).

In addition to feeding state-dependent regulation, OGT preserved excitatory transmission during acute glucose elevations. Wild-type neurons maintained stable synaptic properties under high glucose, whereas OGT-knockout neurons exhibited reduced synaptic frequency and amplitude (Fig. 4, *B*–*G*). Given that O-GlcNAc levels in PVNαCaMKII neurons are particularly sensitive to postprandial glucose (17) these acute effects likely converge with slow feeding-driven modulation, together reflecting a role for OGT in adapting excitatory synaptic input to both rapid and prolonged nutrient fluctuations. Thus, the reduced transmission under high glucose in OGT-knockout neurons represents a maladaptive response to nutrient elevation.

The mechanism by which OGT adapts excitatory synaptic plasticity to metabolic fluctuations in PVN^αCaMKII^ is consistent with a mechanism involving AMPA receptor trafficking. Synaptic events were abolished by NBQX, confirming AMPA receptor dependence (Fig. 3*A*). In addition, OGT deletion altered AMPA receptor composition by increasing GluA1 and reducing GluA2/3 at the surface (Fig. 3, *B*–*J*). Given previous reports that OGT regulates AMPA receptor abundance and synaptic strength (17, 22), these subunit-specific changes provide a molecular basis for the inverted feeding-state response observed in OGT-knockout neurons.

This mechanism supports a model in which OGT represents a fundamentally different class of nutrient sensor compared to canonical glucose-sensing mechanisms in the hypothalamus. While the canonical GLUT2/glucokinase/K_ATP_ channel pathway primarily respond to glucose with an on-off effect on neuron excitability, OGT also influences excitability but, through regulating synaptic plasticity in PVN^αCaMKII^ neurons, makes satiation dependent on upstream network input. Anatomical mapping studies have reported that the PVN receives input from not only other hypothalamic nuclei but also from regions including the amygdala, BNST, hippocampus (35, 36, 37), and indirectly from neocortex (38, 39), raising the possibility that OGT links metabolic state to emotional or cognitive aspects of feeding.

From a behavioral perspective, the feeding state-dependent regulation of excitatory synaptic transmission by OGT uncovers a mechanism by which OGT deletion in PVN^αCaMKII^ in adult mice leads to hyperphagia and impaired meal termination (17). Importantly, OGT’s regulation of synaptic plasticity enables PVN^αCaMKII^ neurons to flexibly reconfigure and prioritize their upstream inputs according to the metabolic context—a functional capacity missing from classical glucose sensors. When OGT is knocked out, neuronal excitability is heightened; however, the inability to selectively filter and integrate incoming signals results in abnormal information processing and a breakdown in proper satiation signaling. This failure to appropriately upregulate and stabilize excitatory signaling in response to nutritional cues directly inhibits satiation and leads to impaired meal termination (40). Consequently, the disruption of energy intake regulation can manifest as excessive consumption, contributing to obesity (5, 6, 41). Conversely, PVN^αCaMKII^ stimulation decreases food intake, and may also be associated with insufficient consumption, characteristic of anorexia nervosa (42), depending on the broader neural circuit context and compensatory mechanisms (43). Taken together, these mechanisms provide a plausible foundation for understanding how metabolic dysfunction can contribute to unhealthy eating behaviors.

Overall, our data support a model in which OGT integrates metabolic information with synaptic machinery to ensure reliable excitatory neurotransmission in PVN^αCaMKII^ neurons across feeding and glucose fluctuations. This adaptive role provides a mechanistic basis towards understanding how the hypothalamus regulates satiation. These observations not only advance our mechanistic understanding of satiation signaling but also highlight how the brain integrates metabolic information to guide complex behavior, offering new perspectives on the neural basis of energy homeostasis and its dysregulation in disease.

## Experimental procedures

### Animals and ethical approval of the study

Five adult male and five female C57BL/6J and OGT-KO mice were housed at the Umeå Center for Comparative Biology at 21 ± 2 °C, under a reversed light cycle (lights off at 23:00 h and on at 11:00 h). Food and water were provided *ad libitum*. All experimental procedures were approved and conducted in accordance with the regulations of the Local Animal Ethics Committee at Umeå University.

Prior to conducting ex vivo experiments, animals were equally divided into two groups: 10 mice in the 24-h starved group and 10 in the non-starved group. The 24-h starved animals were starved for 24 h prior to perfusion, with free access to water. The non-starved animals had *ad libitum* access to food and water.

### Stereotactic virus injection

Anesthesia was induced using isoflurane gas inhalation, and anesthetic depth was maintained at approximately 1.5% isoflurane. Anesthesia depth was monitored by regularly testing pinch reflexes, and core temperature of the animal was maintained using a rectal probe. The coordinates for the PVN were as follows: distance from Bregma, −0.80 mm; distance from midline, ±0.20 mm; depth from surface, −4.80 mm. Animals were placed in a mouse stereotaxic head holder (WPI). The skin was disinfected with Betadine, and a local anesthetic (Marcain 2.5 mg/ml) was applied before incision to expose the skull. Bregma and Lambda were identified, and a cranial window was drilled over the injection site. The viruses pAAV-hSyn-DIO-EGFP (Addgene plasmid #50457; RRID:Addgene_50457) and pENN.AAV.CaMKII0.4.Cre.SV40 (Addgene plasmid #105558; RRID:Addgene_105558) were thawed, and a cocktail was created and diluted with sterile saline to two different final concentrations: 10× and 20×, respectively. The virus was then loaded into a pulled glass pipette (20–40 μm) and connected to a syringe pump (Nanoject III, Drummond Scientific). In total, bilateral injections of 500 nl each of the cocktail virus with 10× dilution were injected into 10 mice (5 WT and 5 OGT-KO), and 500 nl each of the 20× dilution were injected into another 10 mice (5 WT and 5 OGT-KO). The glass capillary was left in place for 5 min before being withdrawn. At the end of surgery, the skin was sutured, and the animals were given a subcutaneous injection of Carprofen (50 mg/ml) to relieve pain.

### Body weight and food intake assessment

Body weight and food intake were measured every alternate day after 10 days following stereotactic surgery, up to a maximum 6 days before tissue harvest. Mice were individually housed with free access to food and water. Body weight was recorded each morning using a precision balance. Food consumption was determined by weighing the food hopper at the same time each day and subtracting residual food weight to calculate daily intake. Data were averaged over the monitoring period for each animal with a maximum gap of 6 days before harvest. Statistical comparisons between WT and OGT-KO groups were performed using the Mann–Whitney non-parametric test, with significance set at *p* < 0.05.

### Immunohistochemistry

Mice were euthanized by cervical dislocation and immediately perfused intracardially with chilled artificial cerebrospinal fluid (aCSF). Serial brain sections were then cut using a microtome. Free-floating sections were blocked in blocking buffer (5% normal goat serum and 0.25% Triton X-100 in PBS) overnight at 4 °C on a shaker. Afterward, they were incubated with primary antibodies overnight at 4 °C on a shaker, and subsequently with secondary antibodies for 2 h on a shaker at room temperature. DAPI was included with the secondary antibodies. Primary and secondary antibodies were diluted in the blocking buffer. After both the primary and secondary antibody incubations, washes were performed using 0.25% Triton X-100 in PBS. The primary antibodies used were GFP (Thermo Fisher Scientific, cat. no. AB_2942817, dilution 1:1000) and OGT (Proteintech, cat. no. 11576-2-AP, dilution 1:1000). The secondary antibodies used were anti-GFP (Goat anti-chicken secondary antibody, AzureSpectra 490, cat. no. AC2209, dilution 1:1000) and anti-OGT (Goat anti-rabbit secondary antibody AzureSpectra 650, cat. no. AC2165, dilution 1:1000). After the final washes, sections were mounted on glass microscope slides and cover slipped using Invitrogen Fluoromount-G Mounting Medium. Slides were dried overnight at room temperature in the dark.

### Imaging analysis

Imaging was performed using a Leica SP8 confocal laser scanning microscope, and the same acquisition settings were maintained to analyse the paraventricular nucleus (PVN) in each WT and OGT-KO section. In the PVN, αCaMKII neurons were identified as cells expressing DAPI, GFP, and OGT signals in WT sections, and DAPI and GFP signals in OGT-KO sections. Images were processed and analysed using Fiji (ImageJ). The cell body diameter of each αCaMKII neuron was measured to classify them as either parvocellular or magnocellular neurons.

### Tissue preparation

Mice were euthanized by cervical dislocation without anaesthesia, followed by a quick transcardial perfusion with a Choline-Chloride (ChCl)-based, ice-cold cutting solution, saturated with 100% oxygen containing (in mM): 110 ChCl, 26 NaAc, 2.5 Glucose, 11.6 Na-Ascorbate, 3.1 Na-Pyruvate, 10 HEPES, 2.5 KCl, 7 MgCl_2_, 0.5 CaCl_2_. This method was used to quickly cool down brain tissue and improve tissue survivability.

The brain was extracted and placed in the same ice-cold ChCl solution. Frontal and caudal ends of the brain were removed, and the remaining brain was placed in a 752M Vibroslicer (Campden Instruments Limited). Coronal slices of 300 μm thickness containing PVN were obtained and incubated at 28 °C in low glucose aCSF saturated with 100% oxygen, containing (in mM): 125 NaCl, 2.5 KCl, 26 NaAc, 2 MgCl_2_, 2 CaCl_2_, 10 HEPES, 2.5 glucose, 17.5 sucrose.

### Electrophysiological recordings

PVN^αCaMKII^ neurons were identified *via* fluorescence using a monochromator controlled by TILLVisIon software (T.I.L.L. Photonics GmbH Munich). GFP-positive cells were selected for electrophysiological recordings. Conventional patch-clamp recordings were performed using borosilicate micropipettes (Harvard Apparatus), made using a Flaming/Brown micropipette puller (Model P-97, Sutter Instrument CO) to a resistance of 3 to 4 MΩ when submerged in the bath. Micropipettes were back-filled with a 0.2% biocytin intracellular solution containing (in mM): 110 K-gluconic acid, 10 NaCl, 1 MgCl_2_, 10 EGTA, 40 HEPES, 2 Mg-ATP, and 0.3 Na-ATP.

Excitatory post-synaptic currents were recorded in voltage-clamp (VC) mode at a holding voltage of −53 to −65 mV, and it was corrected for liquid junction potential. Liquid junction potential was calculated according to the stationary Nernst–Planck equation (44) using LJPcalc (RRID:SCR_025044). Basic electrical properties of the neurons were measured in current-clamp (CC) mode, injecting currents in a sequential manner to detect the different thresholds for action potential and LTS, as well as to calculate neuron excitability.

Protocol began with CC recordings for Vm, AP threshold and LTS measurement, followed by a VC recording of sEPSCs for 5 min. In a subset of experiments, the AMPA receptor antagonist NBQX (10 μM) (Cayman chemicals; 38281) was bath-applied to confirm that spontaneous excitatory post-synaptic currents were AMPA receptor-mediated. Each set of protocols lasted approximately 10 min. The first two sets of recordings were performed under low (2.5 mM) glucose conditions. During the third VC recording, bath application of high (12.5 mM) glucose was initiated after 1 minute. The protocol continued in the same manner in high glucose for up to 30 min. Cells that survived the complete protocol were photographed and used to measure their size and compare with their respective capacitance to corroborate the selection of parvocellular neurons. Recordings represent individual neurons rather than mouse averages; across 10 mice, 6 neurons displayed LTS characteristics, while 10 neurons did not.

### Lentivirus production

To generate lentiviral constructs for OGT knockout in cultured neurons, pseudotyped vesicular stomatitis virus G (VSV-G) lentiviruses expressing either GFP alone (wild-type control) or GFP together with Cre recombinase (OGT knockout) were produced following standard protocols. HEK293T cells were co-transfected with the transfer vector (FUGW or FUGW-Cre), the packaging plasmid (pCMVΔR8.9), and the envelope plasmid (pMD2.G-VSV-G) using Lipofectamine 2000 (Invitrogen, #12313563) according to the manufacturer’s instructions. Viral supernatants were collected 48 to 64 h post-transfection, filtered through 0.45 μm filters, and concentrated by ultracentrifugation. Viral aliquots were stored at −80 °C until use.

### Primary neuronal culture

Primary cortical and hippocampal neurons were prepared from embryonic day 16.5 (E16.5) Ogtˆfl/flˆ or wild-type littermate mice, as described previously (17, 22, 45). Forebrains were dissected in ice-cold Hank’s balanced salt solution (HBSS) and enzymatically dissociated with papain (20 U/ml; Worthington) for 20 min at 37 °C. Cells were gently triturated in NM5 (Neurobasal medium (Gibco, #11570556) supplemented with 2% (v/v) B27 (Invitrogen, #15360284), 2 mM GlutaMAX (Gibco, #35050–038), 5% (v/v) fetal bovine serum (FBS) (Cytiva, #10309433), and 100 U/ml penicillin/streptomycin(Pen-Strep) (Gibco, #15140–122).

Dissociated cortical neurons were plated on poly-D-lysine– or poly-L-lysine–coated 35 mm dishes or coverslips at a density of 1 × 10^6^ cells per well. After 2 h, the plating medium (NM5) was replaced with fresh NM5 medium. Cultures were maintained at 37 °C in a humidified incubator (5% CO_2_). At DIV3–5, glial proliferation was suppressed by adding 5 μM 5-fluoro-2′-deoxyuridine (FDU). From this point on, half of the medium was replaced every 3 to 4 days with glia-conditioned Neurobasal medium containing 1% FBS and 2% B27. For genetic manipulation, cortical neurons were transduced at DIV2 with lentiviral vectors encoding Cre-GFP (for OGT knockout) or GFP alone (control). Cortical neurons were used between DIV14 and DIV18.

### Surface biotinylation and pulldown

At 14 to 18 days *in vitro* (DIV14–18), cultured neurons were washed three times with ice-cold aCSF (143 mM NaCl, 5 mM KCl, 2 mM CaCl_2_, 1 mM MgCl_2_, 10 mM HEPES, pH 7.4- and 10-mM D-glucose). Surface proteins were labeled by incubating cells on ice for 20 min with 1 mg/ml Sulfo-NHS-SS-Biotin (Fisher Scientific, 15332627) prepared in aCSF. Excess biotin was quenched twice with 50 mM glycine in aCSF for 5 min each. Cells were then lysed in neural lysis buffer containing 50 mM Tris–Cl (pH 7.4), 150 mM NaCl, 1% (v/v) Triton X-100, and 0.1% (w/v) SDS, supplemented with protease, phosphatase, and O-GlcNAcase inhibitors. Lysates were clarified by centrifugation at 14,000*g* for 10 min at 4 °C, and the supernatants were incubated overnight at 4 °C with NeutrAvidin agarose beads (Thermo Fisher Scientific, 11885835) to isolate biotinylated surface proteins. Beads were washed three times with neural lysis buffer and eluted with 2× Laemmli sample buffer containing 50 mM DTT by heating at 45 °C for 10 min, as recommended by the antibody manufacturer to prevent protein aggregation. Eluted samples were analyzed by SDS–PAGE and Western blotting.

### Western blot

Homogenized brain tissue was lysed in neural lysis buffer containing 50 mM Tris–Cl (pH 7.4), 150 mM NaCl, 1% (v/v) Triton X-100, and 0.1% (w/v) SDS, supplemented with protease, phosphatase, and O-GlcNAcase inhibitors. Lysates were cleared by centrifugation at 13,000 rpm for 15 min at 4 °C, and the supernatants were collected for protein analysis. Protein samples were mixed with loading buffer and denatured by boiling for 10 min at 90 °C.

Western blotting was performed using standard procedures. Equal amounts of protein were separated by SDS–PAGE and transferred onto polyvinylidene fluoride (PVDF) membranes. Membranes were blocked in 5% non-fat milk prepared in TBS-T and incubated overnight at 4 °C with primary antibodies against OGT (Proteintech, 11576-2-AP; 1:2000), HSP70 (Proteintech, 10995-1-AP; 1:10,000), GluA1 (NeuroMab, N355/1; 5μg/blot), GluA2 (NeuroMab, L21/32; 5μg/blot), or GluA3 (Almone labs, AGC-010-GP; 1:1000). After washing, membranes were incubated with HRP-conjugated secondary antibodies (anti-rabbit, Thermo Fisher Scientific, 31460; or anti-mouse, or anti-guinea pig, Invitrogen, A18769) for 1 h at room temperature. Protein bands were visualized using enhanced chemiluminescence detection reagent (Thermo Fisher Scientific, 34580) and imaged with a Sapphire Biomolecular Imager (Azure Biosystems, IS4000). Band intensities were quantified using Image J software and normalized to HSP70 as a loading control for homogenate(total) samples.

### Data analysis

Frequency, amplitude and decay tau were calculated from VC recordings in a semi-automated manner using MiniAnalysis software (Synaptosoft), whereas Vm, action potential threshold, resistance and LTS were calculated from CC files using Clampfit 11.2 (Molecular Devices, LLC.).

Statistics were performed using Origin 2021b (OriginLab Corporation) and GraphPad Prism 8. Mann-Whitney non-parametric test was used for comparing non-paired samples, while Wilcoxon signed rank test was used for paired samples. Statistical significance was set at a *p*-value <0.05.

Researchers involved in electrophysiological recordings and analysis were blinded during the data acquisition and analysis, revealing the genotype and feeding status of the animal only when the analysis was completed. This method was used to prevent any bias towards either condition, maintaining complete neutrality.

### Schematic representation

Figures 1*A* and 5 were generated using BioRender.com.

## Data availability

All data supporting the findings of this study are included within the article. Raw electrophysiological recordings, uncropped Western blot images, and quantified datasets are available from the corresponding author upon reasonable request.

## Conflict of interest

The authors declare that they do not have any conflicts of interest with the content of this article.
